# Mesenchymal stem cell-derived secretome enhances nucleus pulposus cell metabolism and modulates extracellular matrix gene expression *in vitro*

**DOI:** 10.3389/fbioe.2023.1152207

**Published:** 2023-03-16

**Authors:** Veronica Tilotta, Gianluca Vadalà, Luca Ambrosio, Claudia Cicione, Giuseppina Di Giacomo, Fabrizio Russo, Rocco Papalia, Vincenzo Denaro

**Affiliations:** ^1^ Laboratory for Regenerative Orthopaedics, Research Unit of Orthopaedic and Trauma Surgery, Department of Medicine and Surgery, Università Campus Bio-Medico di Roma, Rome, Italy; ^2^ Operative Research Unit of Orthopaedic and Trauma Surgery, Fondazione Policlinico Universitario Campus Bio-Medico, Rome, Italy

**Keywords:** low back pain, intervertebral disc degeneration, intervertebral disc, secretome, mesenchymal stromal cells, growth factors

## Abstract

**Introduction:** Intradiscal mesenchymal stromal cell (MSC) therapies for intervertebral disc degeneration (IDD) have been gaining increasing interest due to their capacity to ameliorate intervertebral disc metabolism and relieve low back pain (LBP). Recently, novel investigations have demonstrated that most of MSC anabolic effects are exerted by secreted growth factors, cytokines, and extracellular vesicles, collectively defined as their secretome. In this study, we aimed to evaluate the effect of bone-marrow-MSCs (BM-MSCs) and adipose-derived stromal cells (ADSCs) secretomes on human nucleus pulposus cells (hNPCs) *in vitro*.

**Methods:** BM-MSCs and ADSCs were characterized according to surface marker expression by flow cytometry and multilineage differentiation by Alizarin red, Red Oil O and Alcian blue staining. After isolation, hNPCs were treated with either BM-MSC secretome, ADSC secretome, interleukin (IL)-1β followed by BM-MSC secretome or IL-1β followed by ADSC secretome. Cell metabolic activity (MTT assay), cell viability (LIVE/DEAD assay), cell content, glycosaminoglycan production (1,9-dimethylmethylene blue assay), extracellular matrix and catabolic marker gene expression (qPCR) were assessed.

**Results:** 20% BM-MSC and ADSC secretomes (diluted to normal media) showed to exert the highest effect towards cell metabolism and were then used in further experiments. Both BM-MSC and ADSC secretomes improved hNPC viability, increased cell content and enhanced glycosaminoglycan production in basal conditions as well as after IL-1β pretreatment. BM-MSC secretome significantly increased *ACAN* and *SOX9* gene expression, while reducing the levels of *IL6*, *MMP13* and *ADAMTS5* both in basal conditions and after *in vitro* inflammation with IL-1β. Interestingly, under IL-1β stimulation, ADSC secretome showed a catabolic effect with decreased extracellular matrix markers and increased levels of pro-inflammatory mediators.

**Discussion:** Collectively, our results provide new insights on the biological effect of MSC-derived secretomes on hNPCs, with intriguing implications on the development of cell-free approaches to treat IDD.

## 1 Introduction

Low back pain (LBP) is one of the most common musculoskeletal symptoms, affecting two-thirds of adults in the world population at least once in their lives. According to the Global Burden of Disease Study, LBP is the first cause of disability worldwide, with significant socioeconomic repercussions (Wu et al., 2020). Indeed, a recent global report estimated that the inevitable impact on employment status, prolonged hospitalization and increased outpatient visits associated with LBP may exceed $100 billion per year in the sole United States (Russo et al., 2021).

The main cause of chronic LBP is intervertebral disc degeneration (IDD): a chronic, progressive process hallmarked by catabolic events such as gradual resident cell loss due to accelerated apoptosis and senescence, extracellular matrix (ECM) breakdown, and tissue inflammation (Vadalà et al., 2019). These events have been hypothesised to primarily occur within the nucleus pulposus (NP): the inner, highly hydrated, gelatinous part of the intervertebral disc (IVD), physiologically providing load-bearing and shock-absorbing properties. During IDD, the NP metabolism drastically shifts towards catabolism, with increased expression of proteolytic enzymes, such as matrix metalloproteinases (MMPs) and a disintegrin and metalloproteinase with thrombospondin motifs (ADAMTS), resulting in glycosaminoglycan (GAG) depletion and progressive tissue dehydration (Le Maitre et al., 2007). This is often accompanied by the increased release of pro-inflammatory cytokines, including tumor necrosis factor (TNF)-α, interleukin (IL)-1β, and IL-6, which further upregulate catabolic processes, stimulate angiogenesis as well as neurotrophic factors, eventually boosting LBP (Risbud and Shapiro, 2014).

In the last decade, the possibility to restore the NP cell population through cell therapy has gathered significant attention. Indeed, intradiscal cell transplantation, especially involving mesenchymal stromal cells (MSCs), has been shown promising in both preclinical and clinical studies (Vadala et al., 2021). Indeed, MSCs can be harvested by diverse sources (such as the bone marrow and adipose tissue), and are able to differentiate towards the NP cell phenotype as well as release growth factors and cytokines, support resident cell metabolism and recruit local progenitor cells to induce endogenous repair of degenerated IVDs (Sakai and Andersson, 2015). However, the hostile microenvironment of IDD, characterized by hypoxia, low glucose, acidic pH, hyperosmolarity, inflammation, and mechanical overload may compromise the survival of MSCs and thus their regenerative capabilities (Loibl et al., 2019).

In this regard, the secretome derived from MSCs may be a cell-free therapeutic tool able to exert many of the properties that have been described for MSC-based treatments. More specifically, the secretome is a “cocktail” of several factors including extracellular vesicles (EVs) (Kumar et al., 2019). The composition of MSC secretome has been thoroughly investigated and key molecules have been deemed responsible for the regenerative mechanisms of MSCs, including immunomodulation, inhibition of cell death and fibrosis, promotion of tissue remodelling and cell recruitment. Interestingly, *in vitro* preconditioning of MSCs with different factors such as 3D culture, drugs, inflammatory cytokines, and hypoxia has also been shown to enhance secretome therapeutic potential (Madrigal et al., 2014).

In this study, we hypothesized that MSC-derived secretome may attenuate the degenerative conditions of the harsh IDD microenvironment by acting on several biological processes, such as cell viability, metabolic activity, ECM synthesis, and inflammation. Therefore, the purpose of this study was to compare the potential therapeutic effects of the secretome derived from bone marrow (BM)-MSCs and adipose-derived stem cells (ADSCs) on human primary NP cells (hNPCs) in an *in vitro* 3D model.

## 2 Materials and methods

**FIGURE 1:**
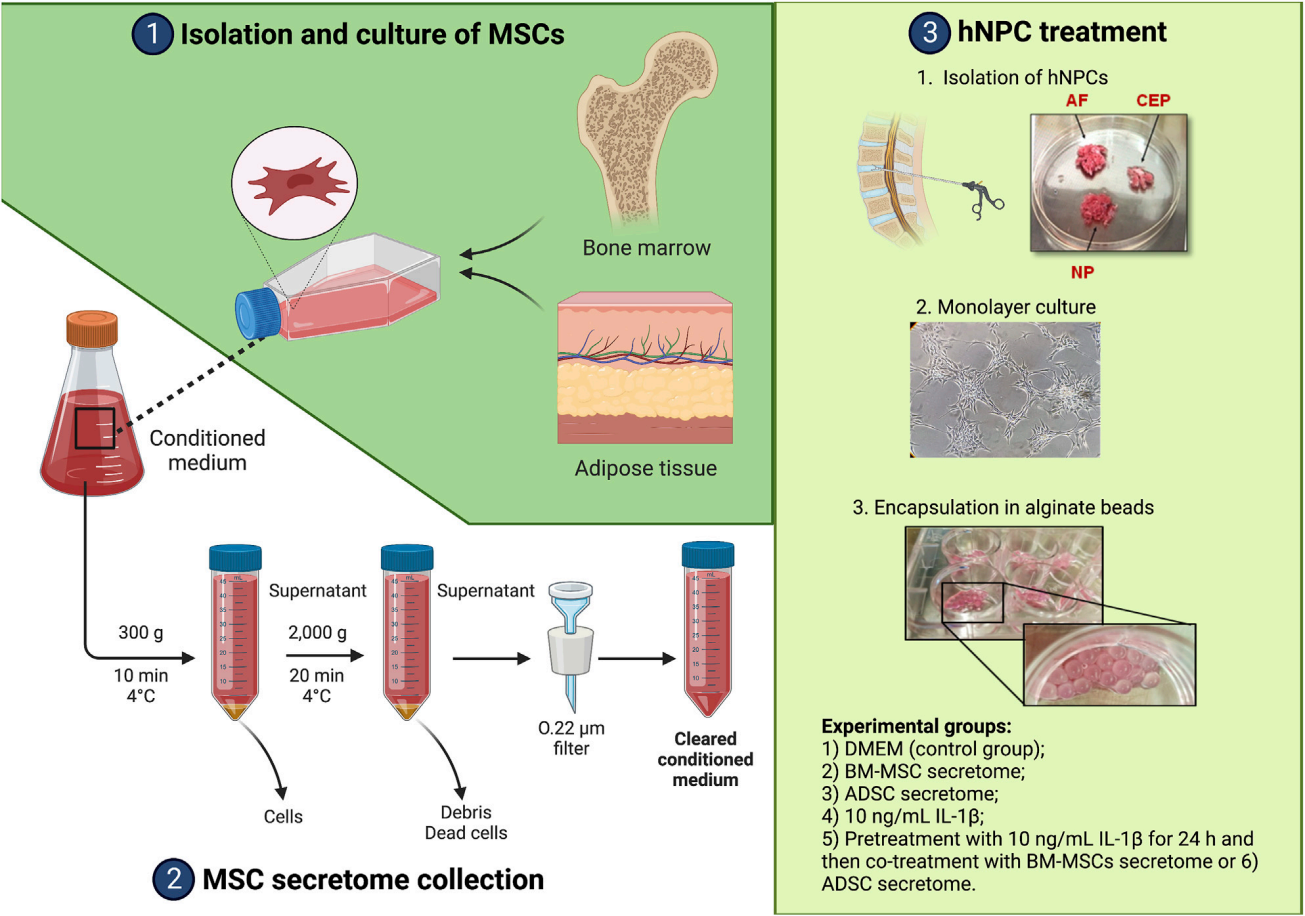
Summary of the experimental workflow. Created with www.biorender.com.

The study was conducted in accordance with the Declaration of Helsinki, and the protocol was approved by the Ethics Committee of Campus Bio-Medico University of Rome (n. 09/15 PAR ComEt CBM). Human IVD and lipoaspirate samples were obtained from surgical waste specimens while BM-MSCs were harvested from healthy donors. Written informed consents were collected from all participants, as described below. A summary of all experimental procedures is depicted in Figure 1.

### 2.1 MSC isolation

BM-MSCs were obtained from 3 healthy donors (M:F = 2:1; mean age = 45 ± 11 years old, no comorbidities) through bone marrow aspiration from the iliac crest using 20 mL-syringes preloaded with 500 IU of preservative-free heparin (PharmaTex Italia, Milano, Italy), as previously described (Cosenza et al., 2018). Cells were incubated in sterile Dulbecco’s Modification of Eagle’s Medium (DMEM; Corning, Corning, NY, United States) containing 1% penicillin/streptomycin (P/S; Sigma, St. Louis, MO, United States) and 20% heat-inactivated fetal bovine serum (FBS) at 37°C with 5% humidified CO_2_. ADSCs were isolated from the stromal vascular fraction (SVF) obtained from lipoaspirate (LA) of 3 patients (M:F = 1:3; mean age = 65 ± 8 years old, body mass index: 34.0 ± 7.1 kg/m^2^, no comorbidities) undergoing intraarticular injection of adipose tissue derivatives for treating knee osteoarthritis. Waste aliquots obtained by LA micro-fragmentation or nanofat processing were used for this study as previously described (Cicione et al., 2023). Briefly, LA was washed with phosphate-buffered saline (PBS), and then digested with 2 mg/mL collagenase type II (Worthington, Columbus, OH, United States) for 40 min at 37°C under gentle agitation. Collagenase was blocked by adding FBS. The digested tissue was filtered through a 70-µm cell strainer (BD, Eysins, Switzerland) and the cell suspension was centrifuged at 300 g for 5 min. The cell pellet was resuspended in αMEM (Corning) with 15% FBS and 1% P/S (Sigma), and cultured in humidified atmosphere at 37°C, with 5% CO_2_. Non-adherent cells were removed by changing the culture medium after 1 day of incubation. Adherent cells were allowed to grow until 70%–80% of confluency and passaged up to the fourth passage (P4).

### 2.2 Flow cytometry analysis

BM-MSCs and ADSCs were characterized as previously described according to minimal criteria provided by the International Society for Cell and Gene Therapy (ISCT) (Dominici et al., 2006). The expression of different BM-MSC and ADSC surface markers was determined *via* flow cytometry as previously described (Cicione et al., 2013). Cells were trypsinized and resuspended in PBS at 1 × 10^5^ cells/mL incubated for 2 h at room temperature in the dark with the following fluorescein isothiocyanate (FITC) or phycoerythrin (PE) conjugated antibodies: AntiCD105-FITC (#561443, BD Biosciences, Haryana, India), antiCD90-PE (#561969, BD Biosciences), antiCD45-PE (#560976, BD Biosciences), and antiCD73-FITC (#561254, BD Biosciences). A minimum of 25,000 cell events per assay were acquired using CytoFLEX (Beckman Coulter, Brea, CA, United States).

### 2.3 Multilineage differentiation

Adherent cells at P4 were induced toward adipogenic, osteogenic and chondrogenic differentiation, as previously described (Cicione et al., 2016). Adipogenic medium was prepared by adding to DMEM-low glucose the following: 10% FBS, 1 µM dexamethasone, 0.5 mM 3–isobutyl-1-methylxanthine (IBMX), 10 ug/mL insulin and 100 µM indomethacin. On the other hand, osteogenic medium composition was prepared by adding to DMEM-low glucose (Corning) the following: 10% FBS, 0.1 µM dexamethasone, 0.2 mM ascorbic acid 2-phosphate, and 10 mM glycerol 2-phosphate. With regards to chondrogenic differentiation, cells were digested with trypsin, washed with high glucose-DMEM and were resuspended in Chondrogenic Differentiation Medium (Lonza, Basel, Switzerland) after centrifugation at 300 g for 5 min. After 21 days, cell differentiation was assessed morphologically using Oil Red-O staining for cytoplasmic lipid droplets, Alizarin red staining for mineralized matrix and Alcian blue staining for cartilaginous ECM. The pellets were fixed in 10% neutral buffered formalin and embedded in paraffin and observed after staining as described above.

### 2.4 Secretome collection

Secretome collection was performed as previously described (Liu et al., 2019). BM-MSCs and ADSCs were extensively washed with PBS. Subsequently, fresh DMEM and αMEM without FBS were added, respectively. After 48 h at 37°C, the conditioned medium (CM) was collected and centrifuged at 300 *g* at 4°C for 10 min to remove gross debris, and then at 2000 g at 4°C for 20 min to remove cellular debris, and further filtered through a 0.22 µm filter. Secretome was stored at −80°C until further use.

### 2.5 Isolation and culture of hNPCs

hNPCs were isolated from IVD tissues of patients undergoing discectomy due to lumbar disc herniation (*n* = 5, mean age = 57 ± 17 years old, Table 1) as previously described (Vadalà et al., 2022). Tissue specimens were washed, annulus fibrosus and cartilaginous endplate tissues were carefully removed, and the NP tissue was minced and digested overnight in DMEM (Corning) with 1% P/S (Sigma), 5% FBS, and 0.01% collagenase type II (Worthington). The digest was filtered through a 70-μm pore size nylon mesh, the cells washed, resuspended in DMEM (Corning) with 10% FBS and 1% P/S (Sigma), and incubated at 37°C in a humidified atmosphere of 5% CO_2_. The culture media were changed twice weekly, and cultures were allowed to grow until reaching 80%–90% confluency. Adherent hNPCs at P2 were treated with trypsin-EDTA (Corning), washed, and centrifuged at 1,500 rpm for 5 min. Then, cell mixtures were resuspended in 1.2% low-viscosity sterile pharmaceutical grade alginate (Pronova Biopolymer, Drammen, Norway) solution at 4 × 10^6^ cells/mL using a syringe with a 21-gauge needle into a 102 mmol/L calcium chloride solution to form semisolid beads (Guerrero et al., 2021). After 10 min of polymerization, beads were washed. The culture was maintained in DMEM (Corning) with 10% FBS and 1% P/S (Sigma) in 5% CO_2_ and 95% air incubator. The media were changed twice a week. hNPCs were then divided in six groups: 1) DMEM (control group); 2) BM-MSC secretome; 3) ADSC secretome; 4) 10 ng/mL IL-1β (Gibco, Grand Island, NY, United States); 5) pretreatment with 10 ng/mL IL-1β for 24 h and then co-treatment with BM-MSCs secretome or 6) ADSCs secretome. Each treatment was performed in triplicate.

**TABLE 1:** Demographics and comorbidities of NP tissue donors.

Age	Sex	Comorbidities
40	M	—
55	M	—
65	M	Hypertension, dyslipidemia
74	M	Hypertension, dyslipidemia, type II DM, CAD
51	M	—

CAD, coronary artery disease; DM, diabetes mellitus; NP, nucleus pulposus.

### 2.6 hNPC metabolic activity

To determine the optimal dose of MSC secretome to be utilized in the experiments, we performed a dose-response curve of MSC-derived CM at different dilutions with normal medium (20%, 40%, 80%, and 100%) by evaluating hNPC metabolic activity through MTT assay (Sigma), according to the manufacturer’s instructions. At P2, hNPCs were seeded in wells of 96-well plates (5 × 103 cells/well). After 48 h, cells were treated with BM-MSC and ADSC secretomes at increasing concentrations and then supplemented with 100 μL of MTT (3-(4,5-Dimethylthiazol-2-yl)-2,5-diphenyltetrazolium bromide) reagent (c = 5 mg/mL). After 4 h at 37°C, MTT solution was removed and 100 μL DMSO were added for 15 min at 37°C to dissolve the formazan crystals. Absorbance readings of DMSO extracts were performed at 570 nm with reference at 690 nm using a TecanInfinite M200 PRO.

### 2.7 hNPC viability and proliferation

Cell viability was determined by analyzing membrane integrity through the LIVE/DEAD™ cell viability assay (Thermo Fisher Scientific, Waltham, MA, United States), following manufacturer’s instructions. After 7 days, hNPCs in alginate beads were incubated for 45 min with ethidium homodimer-2 and calcein acetoxymethylester (AM) at room temperature and washed 3 times with PBS and Hoechst 33258 (Invitrogen, Waltham, MA, United States) to stain the nuclei. Green, red, and blue fluorescence were detected using confocal immunofluorescence microscopy and analyzed with ImageJ. Beads were dissolved by incubation for 10 min at 4°C in 10 volumes of 55 mmol/L sodium citrate, 30 mmol/L EDTA, 0.15 M NaCl, pH 6.8 and cell proliferation was evaluated at 4, 7 and 14 days using CytoFLEX (Beckman Coulter).

### 2.8 GAG production

At the seventh day of the experiment, after dissolving beads, hNPCs were digested with 100 μL of papain (Sigma) solution (0.25 mg/mL in 50 mM phosphate buffer, pH 1.5 containing 5 mM cysteine–hydrochloride and 5 mM ethylenediaminetetraacetic acid) overnight with gentle shaking at 65°C, to assess GAG content. GAGs were measured by reaction with 1,9-dimethylmethylene blue (DMMB; Polysciences, Warrington, PA, United States) using chondroitin sulfate (Sigma) as a standard. Measurements of absorption were performed at a wavelength of 530 nm (Tecan Infinite M200 PRO). Data were expressed as quantity of GAG normalized to DNA content of hNPCs cultured for 7 days, comparing the percent variation between the control group and the experimental group.

### 2.9 RNA extraction and gene expression analysis

After alginate bead digestion, total RNA was extracted from pellets after 7 days of culture using the TRIzol reagent (Invitrogen) according to the manufacturer’s instructions. cDNA was synthetised using the HighCapacity cDNA Reverse Transcription kit (Applied Biosystems, Foster City, CA, United States) according to the manufacturer’s instructions. mRNA levels were measured through qRT-PCR using TaqMan Gene Expression Assays and TaqMan Universal Master Mix II (Thermo Fisher Scientific) with UNG-Real Time PCR System Instrument 7900HT FAST according to manufacturer’s instructions. *ACAN* (Hs0153936), *MMP-13* (Hs00233992), *ADAMTS*5 (Hs00199841), *SOX9* (Hs01001343), and *IL6* (Hs00174131) gene expression were analyzed, and glyceraldehyde 3-phosphate dehydrogenase (*GAPDH*) (Hs03929097) was used as a housekeeping gene. The expression level of each gene has been normalized to the expression of GAPDH and calculated as 2^−ΔDCt^. Values in the experimental group were normalized to expression levels encountered in the control group, which was considered as a baseline.

### 2.10 Statistical analysis

Quantitative data are expressed as means ± standard deviation (SD). The normality of data distribution was determined with the Wilk-Shapiro test. Statistical analysis of the results was performed using unpaired *t*-test and one-way analysis of variance (ANOVA) with Dunnett’s or Tukey’s post-tests for multiple comparisons whenever applicable. Statistical significance was set as *p* < 0.05. Formal analysis was performed using Prism 9 (GraphPad, San Diego, CA, United States). Each experiment was repeated at least three times and representative experiments are shown.

## 3 Results

### 3.1 Identification of MSCs

**FIGURE 2:**
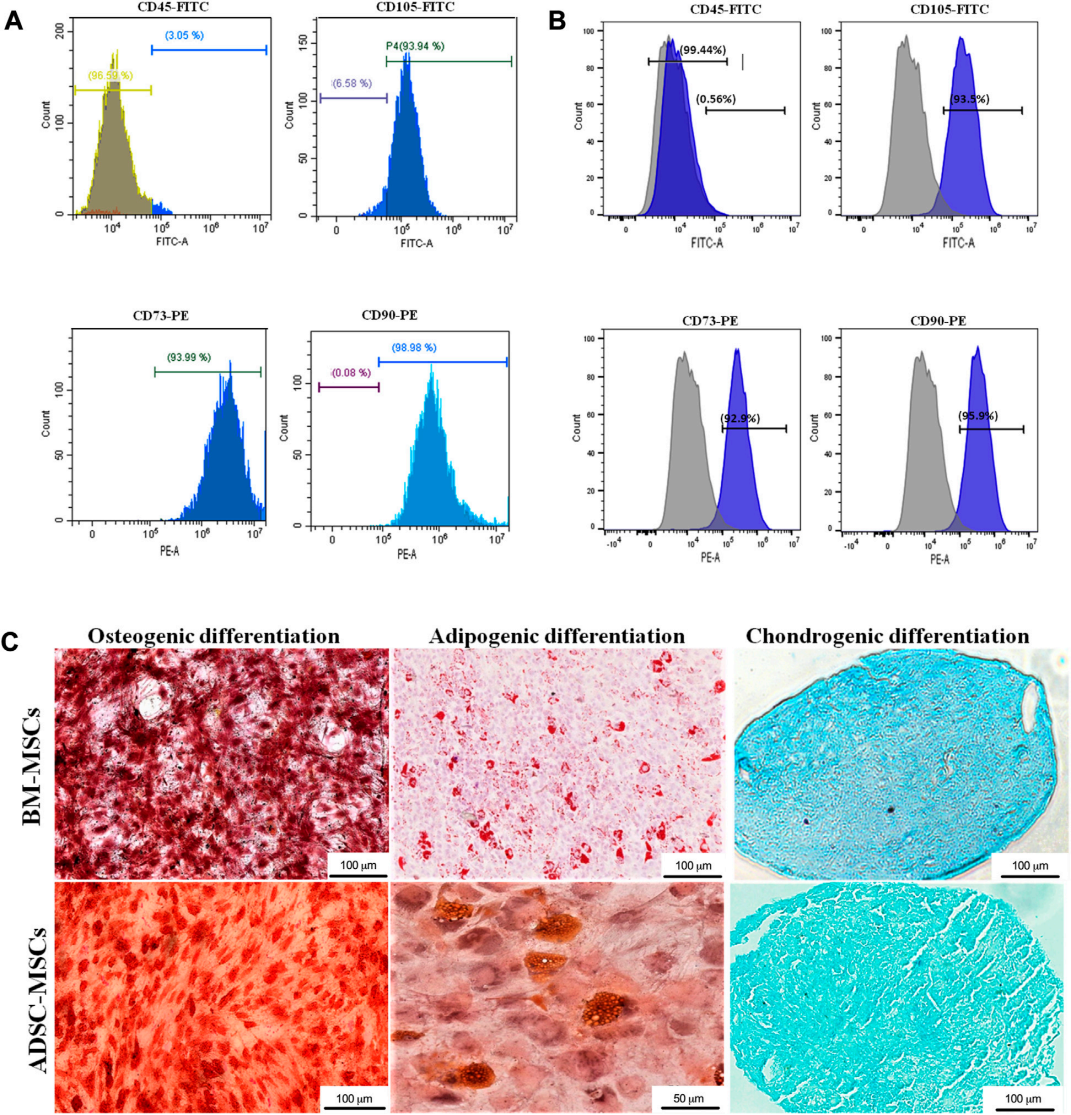
Characterization of MSCs. BM-MSCs **(A)** and ADSCs **(B)** where characterized with flow cytometry through immunophenotype analysis of MSC cell surface markers CD45, CD105, CD73, CD90. **(C)** Osteogenic, adipogenic, and chondrogenic differentiation was determined by Alizarin red, Oil red O and Alcian blue staining, respectively (magnification: ×20, scale bars: 50 μm and 100 μm).

Flow cytometry analysis showed that both BM-MSCs and ADSCs were positive for the expression markers CD73, CD90 and CD105 and negative for CD45 (Figure 2A). Evidence of MSC multipotency was found in their capacity to differentiate towards osteogenic, adipogenic, and chondrogenic lineages under specific differentiation conditions (Figure 2B). Adipogenic differentiation was detected by the presence of lipid vacuoles at Oil Red-O staining. Red-stained calcium deposits demonstrated osteogenic differentiation through Alizarin Red staining. Finally, cartilaginous ECM deposition was confirmed by Alcian Blue staining (Figure 2C).

### 3.2 hNPC metabolic activity

**FIGURE 3:**
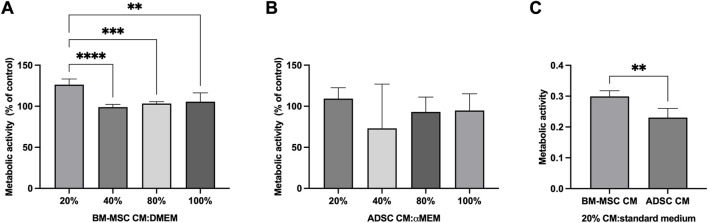
BM-MSC-derived secretome enhanced hNPC metabolic activity. MTT assay on hNPCs treated with BM-MSC-derived CM **(A)** or ADSC-derived CM **(B)** at a 20%, 40%, 80% or 100% concentration (percentage of CM diluted in DMEM, volume/volume) showed that 20% BM-MSC CM yielded the greatest effect towards cell metabolic activity. Data are expressed as percent variation between the control and experimental groups. **(C)** 20% BM-MSC-derived CM determined a significantly higher increase of metabolic activity in hNPCs compared to 20% ADSC-CM. **p < 0.01, ***p < 0.001, ****p < 0.0001.

The effect of MSC secretome towards hNPC metabolic activity was assessed using the MTT assay. hNPCs in the control group were assumed to have 100% mitochondrial activity. A dose-response curve of MSC secretome treatment at different dilutions with normal medium (20%, 40%, 80% 100%) was calculated using control cells as a reference. After 48 h, 20%, BM-MSC secretome significantly increased hNPCs mitochondrial activity (126.3% ± 6.9%) compared to controls (*p* < 0.001) as well as to other secretome concentrations (40%: 98.9% ± 3.3%, *p* < 0.001; 80%: 103.3% ± 2.3%, *p* < 0.001; 100%: 105.5% ± 10.8%, *p* = 0.002; Figure 3A). Conversely, no significant effect of ADSC secretome towards hNPC metabolic activity was encountered at any concentration, although a slight increase was seen following treatment with 20% ADSC secretome (Figure 3B). When comparing 20% BM-MSC and 20% ADSC secretome, the former showed a significantly higher effect on cell metabolism (0.3 ± 0.01 vs. 0.2 ± 0.03, *p* = 0.008; Figure 3C). Therefore, all subsequent experiments were performed using 20% BM-MSC (BM-MSCsec) and ADSC secretomes (ADSCsec).

### 3.3 MSC secretome improved hNPC proliferation and viability

**FIGURE 4:**
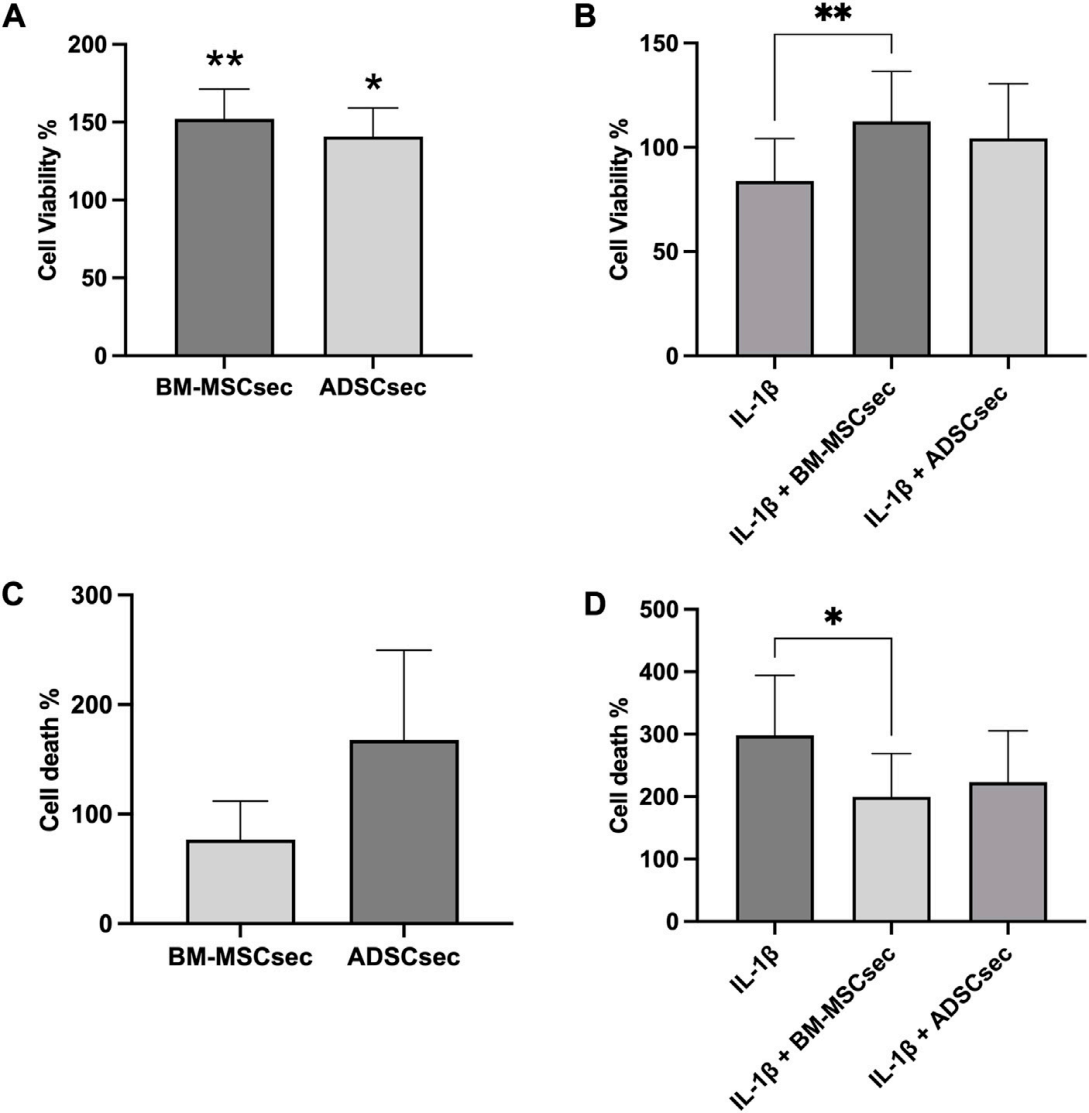
MSC-derived secretome enhanced hNPC viability and reduced cell death. The LIVE/DEAD assay showed that both BM-MSCsec and ADSCsec significantly increased cell viability compared to the control group **(A)**. Following IL-1β pretreatment, BM-MSCsec significantly rescued hNPC vitality compared to cells treated with IL-1β alone **(B)**. **(C)** BM-MSCsec and ADSCsec treatment did not substantially impact on cell death. **(D)** After IL-1β pretreatment, BM-MSCsec significantly reduced the number of dead cells compared to hNPCs treated with IL-1β alone. Results were normalized to the control group and expressed as percent variation. *p < 0.05, **p < 0.01.

**FIGURE 5:**
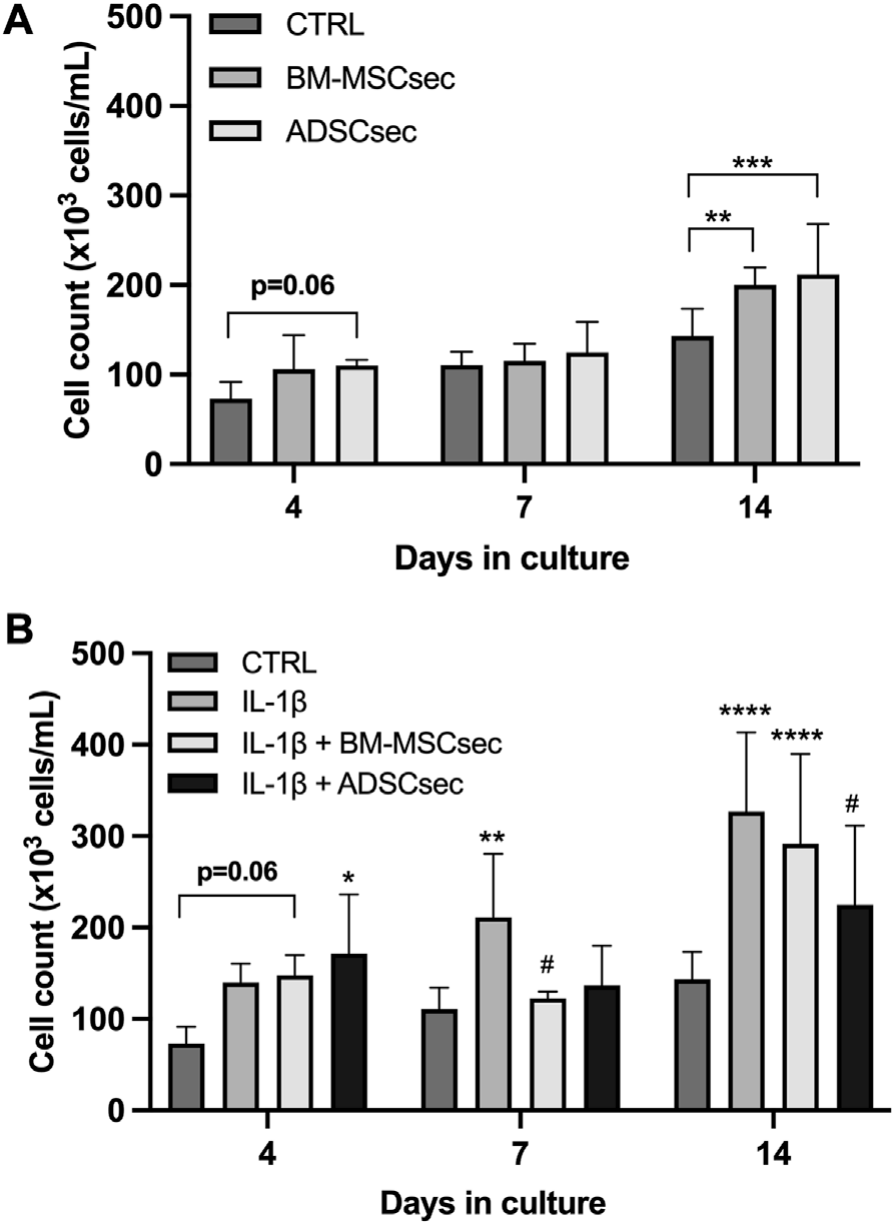
MSC-derived secretome increased hNPC proliferation. **(A)** Both BM-MSCsec and ADSCsec significantly increased hNPC content at 14 days of culture. **(B)** After IL-1β pretreatment, cell numerosity was aberrantly increased at 7 and 14 days compared to the controls. Treatment with BM-MSCsec and ADSCsec resulted in a substantial increase of cell content compared to the control group, while being lower than the group treated with IL-1β. *p < 0.05, **p < 0.01, ***p < 0.001, ****p < 0.0001, ^#^ *p* < 0.05 compared to IL-1β.

hNPCs in alginate beads were cultured for 7 days and stained for live and dead cells as described above (*n* = 4). Results were normalized to the control group and expressed as percent variation. In basal conditions, both BM-MSCsec and ADSCsec were able to significantly increase cell viability (152.1% ± 19.1%, *p* = 0.005 and 140.7% ± 18.3%, *p* = 0.017, respectively; Figure 4A). On the other hand, following IL-1β pretreatment, cell viability dropped to 83.8% ± 20.5% but was significantly increased following treatment with BM-MSCsec (112.5% ± 23.9%, *p* = 0.009) and ADSCsec (104.3% ± 26.2%), even if the latter did not reach statistical significance (Figure 4B). In contrast, cells treated with MeOH for 30 min were assumed to suffer 100% cell death and all other treatments were normalized to this condition. Cells treated with both BM-MSCsec and ADSCsec did not show a significant change of cell death (BM-MSCsec: 76.8% ± 35.0%; ADSCsec: 167.7% ± 82.0%, Figure 4C). Nevertheless, while IL-1β treatment showed a substantial increase of cell death (298.3% ± 95.9%), BM-MSCsec significantly counteracted the detrimental stimulus and reduced hNPC death (199.9% ± 68.9%; *p* = 0.021), while the effect of ADSCsec did not reach statistical significance (223.3% ± 82.5%; Figure 4D). Cell count was performed at different timepoints after digestion of alginate beads. hNPCs (*n* = 4) treated with BM-MSCsec and ADSCsec showed an increase of cell content at 4, 7 and 14 days after treatment compared to the control group (Figure 5A). Such an increase was statistically significant after two weeks in cells treated with both BM-MSCsec (200.0 ± 19.7 × 10^3^ cells/mL, *p* = 0.004) and ADSCsec (211.7 ± 56.5 × 10^3^ cells/mL, *p* < 0.001) compared to the control group (143.3 ± 30.4 × 10^3^ cells/mL). Contrarily, when hNPCs were treated with 10 ng/mL IL-1β, cell numerosity was significantly increased at 7 (211.0 ± 69.4 × 10^3^ cells/mL, *p* = 0.016) and 14 days (326.7 ± 86.8 × 10^3^ cells/mL, *p* < 0.001) compared to the controls (7 days: 110.7 ± 23.5 × 10^3^ cells/mL; 14 days: 143.3 ± 30.4 × 10^3^ cells/mL; Figure 5B). After 4 days, both BM-MSCsec (147.8 ± 21.9 × 10^3^ cells/mL, *p* = 0.06) and ADSCsec (171.5 ± 64.6 × 10^3^ cells/mL, *p* = 0.019) incremented cell content after IL-1β pretreatment compared to the control group (73.0 ± 18.7 × 10^3^ cells/mL). The same trend was reported at day 7 (BM-MSCsec: 122.7 ± 7.4 × 10^3^ cells/mL, *p* = 0.043; ADSCsec: 136.7 ± 43.5 × 10^3^ cells/mL, *p* = 0.016 vs. IL-1β) and 14 (BM-MSCsec: 291.7 ± 98.5 × 10^3^ cells/mL, *p* = 0.002 vs. control; ADSCsec: 225.0 ± 86.6 × 10^3^ cells/mL, *p* = 0.015 vs. IL-1β).

### 3.4 MSC secretome promoted GAG synthesis by hNPCs

**FIGURE 6:**
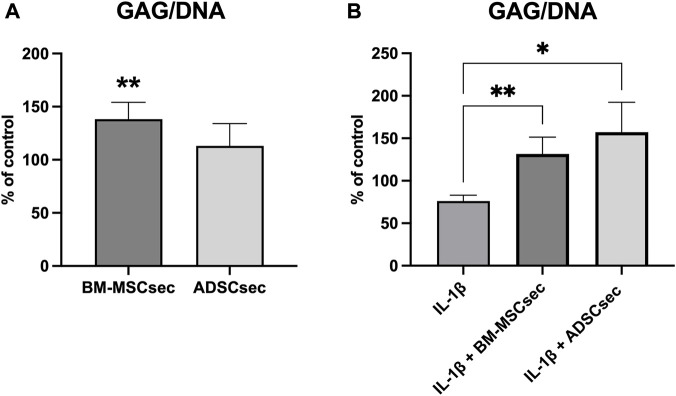
BM-MSCsec increased GAG content in treated hNPCs. **(A)** GAG/DNA ratio in hNPCs after MSC secretome treatment demonstrated a significant increase in the group treated with BM-MSCsec. **(B)** Following IL-1β pretreatment, both BM-MSCsec and ADSCsec demonstrated to rescue GAG/DNA levels compared to the group treated with IL-1β only. Data are expressed as GAG/DNA ratio percent variation between the control and experimental groups. *p < 0.05, **p < 0.01.

Three-dimensional cell cultures (n = 5) treated with BM-MSCsec and ADSCsec showed a significant increase in GAG synthesis normalized to DNA. Considering the GAG/DNA ratio in the control group as a baseline of 100%, hNPCs exposed to BM-MSCsec produced a higher amount of GAG (138.3% ± 15.8%, *p* = 0.009) compared to controls (Figure 6A). Conversely, following IL-1β pretreatment, GAG content was significantly decreased (76.2% ± 6.7%, *p* = 0.005) compared to the control group, but was promptly restored after treatment with both BM-MSCsec (131.5% ± 19.9%, *p* = 0.005) and ADSCsec (157.2% ± 35.2%, *p* = 0.021; Figure 6B).

### 3.5 MSC secretome restored ECM and inflammatory gene expression profile of hNPCs

**FIGURE 7:**
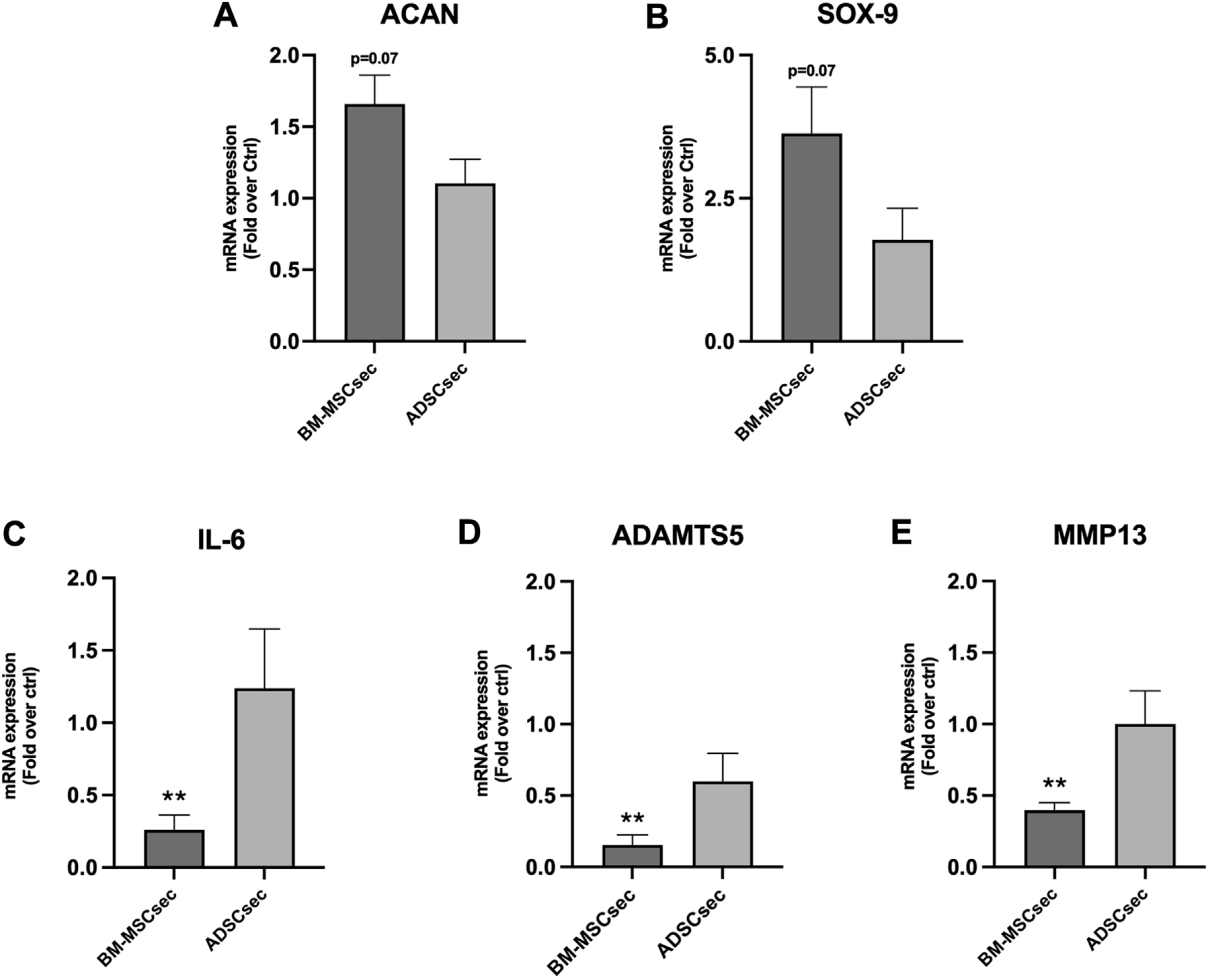
BM-MSCsec improved hNPC ECM and catabolic marker gene expression. hNPCs treated with BM-MSCsec demonstrated a higher expression of *ACAN* **(A)** and *SOX9* **(B)** genes, while significantly reducing *IL6* **(C)**, *ADAMTS5* **(D)** and *MMP13* **(E)** gene expression. Conversely, no significant differences were found in the group treated with ADSCsec. Results were normalized based on *GAPDH* expression and calculated as fold change compared to the controls. **p < 0.01.

**FIGURE 8:**
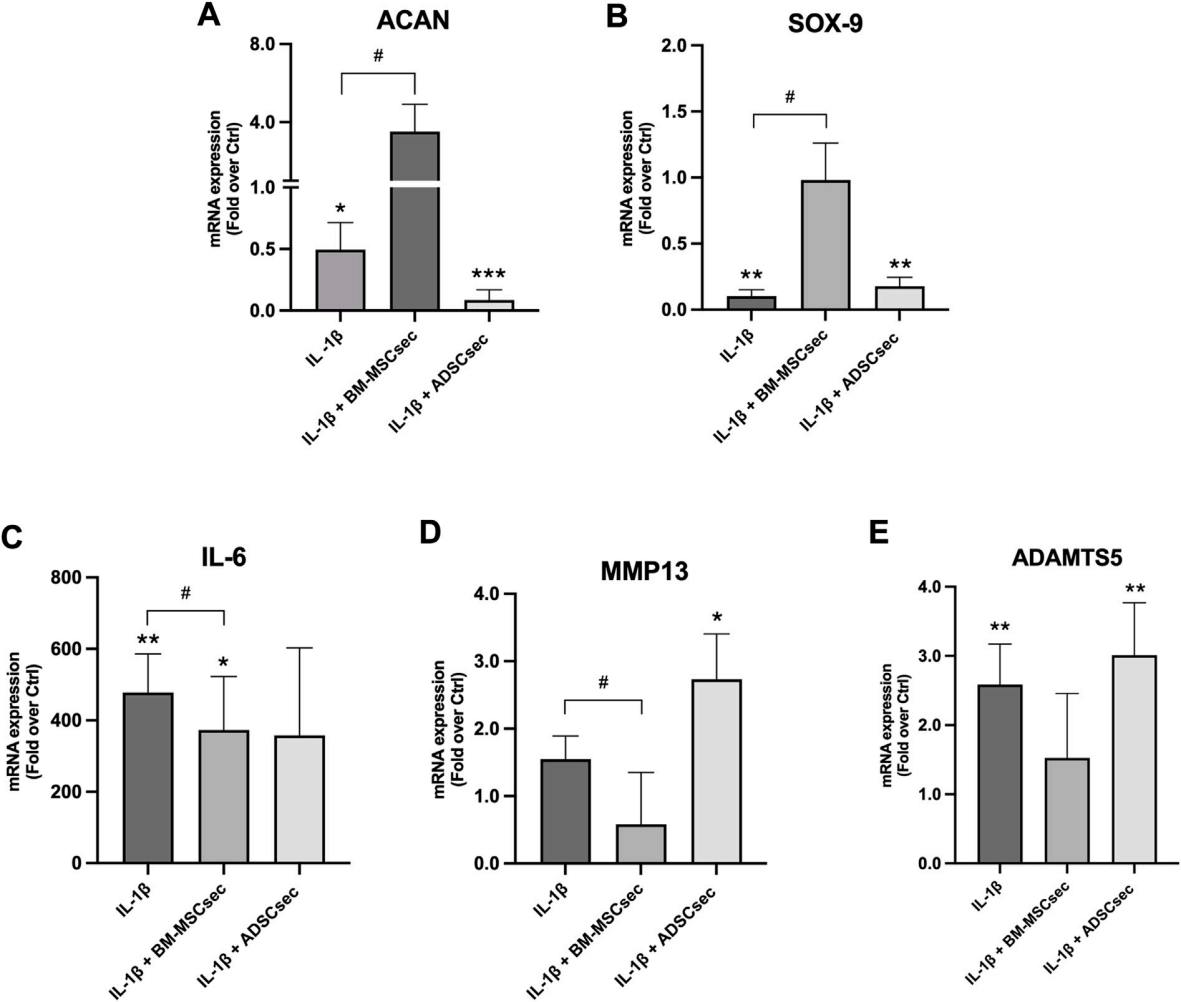
MSC secretomes differentially impacted on hNPC ECM and catabolic marker gene expression following IL-1β pretreatment. BM-MSCsec significantly rescued the expression of *ACAN* **(A)** and *SOX9* **(B)** genes, while significantly reducing *IL6* **(C)**, *ADAMTS5* **(D)** and *MMP13* **(E)** gene expression following IL-1β pretreatment. Conversely, ADSCsec significantly reduced the expression of both *ACAN* and *SOX9*, while increasing catabolic marker levels. Results were normalized based on *GAPDH* expression and calculated as fold change compared to the controls. *p < 0.05, **p < 0.01, ***p < 0.001, ^#^ *p* < 0.05 compared to IL-1β.

After 7 days, MSC secretomes exerted a beneficial effect on inflammatory and catabolic marker gene expression in hNPCs (*n* = 4). More specifically, BM-MSCsec determined an increase of *ACAN* (Figure 7A) and *SOX9* (Figure 7B) gene expression compared to the control group, nearly reaching statistical significance (1.7 ± 0.4, *p* = 0.07; 3.6 ± 1.6, *p* = 0.07, respectively). Furthermore, BM-MSCsec significantly reduced the expression of *IL6* (0.3 ± 0.2, *p* = 0.009; Figure 7C), *ADAMTS5* (0.2 ± 0.1, *p* = 0.002; Figure 7D), and *MMP13* (0.4 ± 0.1, *p* = 0.003; Figure 7E). On the other hand, while showing the same trends, treatment with ADSCsec did not show any statistically significant difference. After pretreating hNPCs with IL-1β, *ACAN* (Figure 8A) and *SOX9* (Figure 8B) gene expression dropped (0.5 ± 0.2, *p* = 0.041; 0.1 ± 0.3, *p* = 0.002, respectively), while increasing *IL6* (478.4 ± 107.6, *p* = 0.006; Figure 8C), *MMP13* (1.6 ± 0.8, *p* = 0.101; Figure 8D) and *ADAMTS5* levels (2.6 ± 0.6, *p* = 0.009; Figure 8E) compared to the control group. Interestingly, the addition of BM-MSCsec rescued both *ACAN* (3.5 ± 1.4, *p* = 0.048 vs. IL-1β) and *SOX9* levels (0.9 ± 0.3, *p* = 0.045 vs. IL-1β), whereas significantly reducing *IL6* (372.9 ± 149.3; *p* = 0.032 vs. IL-1β, *p* = 0.046 vs. control), and *MMP13* gene expression (1.5 ± 0.9, *p* = 0.044 vs. IL-1β). Interestingly, ADSCsec led to a significant decrease of *ACAN* (0.09 ± 0.08, *p* < 0.001) and *SOX9* (0.2 ± 0.07, *p* = 0.006) gene expression, as well as increased levels of *MMP13* (2.7 ± 0.7, *p* = 0.031) and *ADAMTS5* (3.01 ± 0.7, *p* = 0.009) compared to the controls.

## 4 Discussion

The aim of this study was to compare the regenerative potential of secretomes derived from two MSC sources (BM-MSCs and ADSCs) on hNPCs in an *in vitro* 3D construct. Our findings demonstrated that the stimulation of hNPCs with BM-MSCsec and ADSCsec enhanced cell viability and GAG synthesis, attenuated ECM degradation and modulated cell proliferation and inflammation, which are crucial for IVD homeostasis. Furthermore, after pretreating hNPCs with IL-1β to mimic the inflammatory milieu of IDD, MSC-derived secretomes showed to effectively blunt the catabolic effects of *in vitro* inflammation (Le Maitre et al., 2005). Indeed, IDD is characterized by a considerably harsh microenvironment with progressive loss of resident cells and increased ECM breakdown, resulting in an overall shift towards tissue catabolism (Vadalà et al., 2019). Therefore, the use of functional cells to replenish the damaged IVD and possibly hamper degenerative changes has drawn increasing interest in the field. To date, several cell sources have been considered and described for their regenerative potential, with a particular attention towards MSCs, including BM-MSCs, ADSCs and induced pluripotent stem cells (iPSCs) (Vadala et al., 2015; Williams et al., 2021). It is well known that the efficacy of MSCs is mainly mediated by secreted bioactive factors exerting anabolic, proliferative and immunomodulating effect on target cells *via* paracrine mechanisms (Vizoso et al., 2017). In this regard, the identification of secreted factors released in the CM *in vitro* may clarify which are directly involved in the regenerative process, with the possibility to administer or synthesize them artificially as an alternative therapeutic option to the use of transplanted cells (Maffioli et al., 2017). Hingert et al. investigated the content of peptides present in the CM of human MSCs and their effects on both degenerated disc cell and MSC pellets. The results of the mass spectrometry analysis revealed 129 secreted peptides including growth factors, anti-apoptotic and anti-angiogenic factors, and MMP inhibitors (Hingert et al., 2019). Furthermore, factors cointained in MSC secretomes also include EVs, which are involved in cellular communication due to their ability to transport proteins, lipids, and especially nucleic acids. Indeed, EVs can be chemically or biologically modified and selective exosomal mRNAs may “reprogram” target cells by altering or enhancing their physiological state (Tilotta et al., 2021). According to our Live/Dead assay, BM-MSCsec and ADSC-sec were able to increase hNPC viability and decrease cell death compared to the control group. In addition, both were also capable of rescuing hNPCs from the catabolic state induced by IL-1β pretreatment, which led to decreased hNPC vitality and higher cell death rates. Therefore, we hypothesize that MSC-derived secretomes may protect NP resident cells from premature death. In an *in vitro* study, Mehrkens et al. showed that notochordal cell-derived secretome was able to confer anti-apoptotic effects to hNPCs following incubation with IL-1β. Indeed, they reported the inhibition of the cytotoxic activity of caspase-3/7, −8 and −9, as well as maintenance of the mitochondrial membrane potential in hNPCs, thus demonstrating that soluble factors may prevent hNPC apoptosis triggered by the inflammatory stimulus (Mehrkens et al., 2017). MSC-derived secretomes also exerted a notable effect on cell proliferation. In basal conditions, both BM-MSCsec and ADSC-sec increased hNPC numerosity at all timepoints under study, with a statistically significant growth potential at 14 days. Interestingly, after IL-1β pretreatment, a prominent increase of cell proliferation was noted. According to the literature, this phenomenon may be interpreted as a reparative response that can be directly triggered by IL-1β through the activation of the NOTCH pathway, which is also associated with upregulation of catabolic and inflammatory mediators (Wang et al., 2013). However, the addition of BM-MSCsec and ADSC-sec, while increasing cell number compared to the control group, also reduced hNPC numerosity compared to cells treated with IL-1β only, possibly showing an inhibitory effect towards such IL-1β-induced response. The anabolic effect of MSC-derived secretome was also demonstrated by increased GAG levels in both basal and pro-inflammatory conditions. This is in line with previous reports, demonstrating a higher abundancy of GAG and ECM production *in vitro* (Hingert et al., 2020), *ex vivo* (Ferreira et al., 2021) and *in vivo* (Matta et al., 2017) following secretome treatment. Gene expression of healthy NPC phenotypic markers (namely, *ACAN* and *SOX9*), catabolic enzymes involved in ECM degradation (*ADAMTS5* and *MMP13*) and the proinflammatory cytokine *IL6* were assessed to further investigate the biological effect of MSC-derived secretomes. In a recent study by Zeng et al., umbilical cord-derived MSC secretome was shown to significantly increase the expression of *TEK*, a marker of NP progenitor cells, as well as pluripotency markers *OCT4* and *NANOG*. They concluded that MSC-derived secretome may play a key role in the promotion of de-differentiation, stemness and multipotency of degenerated NPCs by highlighting its potential “rejuvenating” effects (Zeng et al., 2020). Interestingly, different responses between BM-MSCsec and ADSCsec were noted in our study. While both showed no substantial increase of *ACAN* and *SOX9* levels in basal conditions, BM-MSCsec significantly reduced the expression of *ADAMTS5*, *MMP13*, and *IL6*. However, following IL-1β pretreatment, BM-MSCsec significantly increased *ACAN* and *SOX9* expression compared to hNPCs treated with IL-1β alone, whereas reducing *IL6* and *MMP13* levels. On the other hand, ADSCsec showed a catabolic effect, characterized by significantly lower levels of *ACAN* and *SOX9* with higher expression of catabolic markers. Indeed, as already described in previous studies, ADSCs have been shown to act as either immune-enhancing and immunosuppressive mediators (Leto Barone et al., 2013). Compared to BM-MSCs, ADSCs are significantly more influenced by donors’ unique characteristics, including previous medical history, drug assumption and metabolic disorders, especially obesity. Collectively, these variables are known to profoundly affect the adipose tissue microenvironment and, consequently, ADSC biological activity and secretory profile (Parsons et al., 2018). Therefore, while showing immunomodulatory and anti-inflammatory effects, previous reports have also displayed the capacity of ADSCs to increase the expression of several pro-inflammatory mediators, including IL-1β, IL-6, IL-8, monocyte chemoattractant protein (MCP)-1, MMP-1, MMP-13, ADAMTS-4, and prostaglandin E2 (PGE_2_) (Parsons et al., 2018; Borem et al., 2019; Cavallo et al., 2022). It is not surprising that, considering that the average body mass index in our cohort of ADSC donors exceeded the obesity threshold (>30 kg/m^2^), ADSCsec exerted a catabolic effect on gene expression. In this regard, since paracrine stimulation has a significant impact on MSC activity, the importance of cell preconditioning to increase the therapeutical potential of their secretome is being increasingly evident (Ferreira et al., 2018). For example, low oxygen levels have shown to improve the survival capacity of MSCs by increasing the release of cytoprotective molecules and favour the maintenance of an undifferentiated state (Romaniyanto et al., 2022). Similarly, culturing MSCs in a 3D environment has demonstrated to enhance the release of anti-inflammatory cytokines and factors involved in tissue regeneration and cell migration compared to cells cultured in monolayer in an *in vivo* model of rheumatoid arthritis treated with umbilical cord MSC-derived secretomes (Petrenko et al., 2017). Furthermore, preconditioning of MSCs with soluble factors and pro-inflammatory cytokines such as IL-1β, TNF-α and interferon (IFN)-γ showed to increase the secretion of immunomodulatory proteins such as indoleamine-pyrrole 2,3-dioxygenase, PGE_2_, and IL-6 (Mancuso et al., 2019). Despite encouraging evidence, data on the effects of MSC preconditioning also display a high degree of heterogeneity in secretome composition (Assoni et al., 2017). This variability can be attributable to different MSC sources, donor age and health status, as well as methods of secretome production.

The study has some limitations. First, we did not select the patients from whom we collected MSCs neither by age nor presence or absence of comorbidities. Second, hNPCs were derived from patients with varying degrees and levels of IDD. This, together with the presence of additional covariates (e.g., sex, age, comorbidities), may have contributed to generate donor variability, although our results did not seem to be significantly affected by substantial heterogeneity. Finally, since MSCs were not preconditioned as mentioned above, the use of different variables may have greatly improved secretome regenerative capacity.

## 5 Conclusion

This study compared the potential beneficial effect of MSC secretome isolated from two different sources, namely, BM-MSCs and ADSCs, on hNPCs cultured in a 3D environment. Both secretomes improved hNPC proliferative capacity, viability, and GAG synthesis both in basal conditions and following pro-inflammatory preconditioning with IL-1β. In terms of gene expression of ECM and inflammatory markers, BM-MSCsec appeared to exert a greater regenerative effect compared to ADSCsec, which in turned showed a catabolic expression profile, further suggesting the importance of selecting the right cell source. Despite the lack of consensus regarding culture conditions as well as standardized protocols for preparation, storage, product stability and quality control parameters, the use of MSC-derived secretome may be a promising cell-free approach for the treatment of IDD.

## Data Availability

The raw data supporting the conclusion of this article will be made available by the authors, without undue reservation.

## References

[B1] Assoni A. Coatti G. Valadares M. C. Beccari M. Gomes J. Pelatti M. (2017). Different donors mesenchymal stromal cells secretomes reveal heterogeneous profile of relevance for therapeutic use. Stem Cells Dev. 26 (3), 206–214. 10.1089/scd.2016.0218 27762666

[B2] Borem R. Madeline A. Bowman M. Gill S. Tokish J. Mercuri J. (2019). Differential effector response of amnion- and adipose-derived mesenchymal stem cells to inflammation; implications for intradiscal therapy. J. Orthop. Res. 37, 2445–2456. 10.1002/jor.24412 31287173

[B3] Cavallo C. Merli G. Zini N. D’Adamo S. Cattini L. Guescini M. (2022). Small extracellular vesicles from inflamed adipose derived stromal cells enhance the NF-κB-Dependent inflammatory/catabolic environment of osteoarthritis. Stem Cells Int. 2022, 1–19. 10.1155/2022/9376338 PMC9314187 35898656

[B4] Cicione C. Di Taranto G. Barba M. Isgrò M. A. D’Alessio A. Cervelli D. (2016). *In vitro* validation of a closed device enabling the purification of the fluid portion of liposuction aspirates. Plastic Reconstr. Surg. 137 (4), 1157–1167. 10.1097/prs.0000000000002014 26741887

[B5] Cicione C. Muiños-López E. Hermida-Gómez T. Fuentes-Boquete I. Díaz-Prado S. Blanco F. J. (2013). Effects of severe hypoxia on bone marrow mesenchymal stem cells differentiation potential. Stem Cells Int. 2013, 1–11. 10.1155/2013/232896 PMC3777136 24082888

[B6] Cicione C. Vadalà G. Di Giacomo G. Tilotta V. Ambrosio L. Russo F. (2023). Micro-fragmented and nanofat adipose tissue derivatives: *In vitro* qualitative and quantitative analysis. Front. Bioeng. Biotechnol. 11, 911600. 10.3389/fbioe.2023.911600 36733959 PMC9887143

[B7] Cosenza S. Toupet K. Maumus M. Luz-Crawford P. Blanc-Brude O. Jorgensen C. (2018). Mesenchymal stem cells-derived exosomes are more immunosuppressive than microparticles in inflammatory arthritis. Theranostics 8 (5), 1399–1410. 10.7150/thno.21072 29507629 PMC5835945

[B8] Dominici M. Le Blanc K. Mueller I. Slaper-Cortenbach I. Marini F. Krause D. (2006). Minimal criteria for defining multipotent mesenchymal stromal cells. The International Society for Cellular Therapy position statement. Cytotherapy 8 (4), 315–317. 10.1080/14653240600855905 16923606

[B9] Ferreira J. R. Teixeira G. Q. Neto e. Ribeiro-Machado c. Silva A. M. Caldeira J. (2021). IL-1β-pre-conditioned mesenchymal stem/stromal cells’ secretome modulates the inflammatory response and aggrecan deposition in intervertebral disc. Eur. Cells Mater. 41, 431–543. 10.22203/eCM.v041a28 33877647

[B10] Ferreira J. R. Teixeira G. Q. Santos S. G. Barbosa M. A. Almeida-Porada G. Goncalves R. M. (2018). Mesenchymal stromal cell secretome: Influencing therapeutic potential by cellular pre-conditioning. Front. Immunol. 9, 2837. 10.3389/fimmu.2018.02837 30564236 PMC6288292

[B11] Guerrero J. Hackel S. Croft A. S. Albers C. E. Gantenbein B. (2021). The effects of 3D culture on the expansion and maintenance of nucleus pulposus progenitor cell multipotency. JOR Spine 4 (1), e1131. 10.1002/jsp2.1131 33778405 PMC7984018

[B12] HingertD.NawilaijaroenP.AldridgeJ.BarantoA.BrisbyH. (2019). Investigation of the effect of secreted factors from mesenchymal stem cells on disc cells from degenerated discs. Cells Tissues Organs 208 (1-2), 76–88. 10.1159/000506350 32092752

[B13] Hingert D. Nawilaijaroen P. Ekström K. Baranto A. Brisby H. (2020). Human levels of MMP-1 in degenerated disks can Be mitigated by signaling peptides from mesenchymal stem cells. Cells Tissues Organs 209 (2-3), 144–154. 10.1159/000509146 32829335

[B14] Kumar P. L. Kandoi S. Misra R. S V. K R. Verma R. S. (2019). The mesenchymal stem cell secretome: A new paradigm towards cell-free therapeutic mode in regenerative medicine. Cytokine Growth Factor Rev. 46, 1–9. 10.1016/j.cytogfr.2019.04.002 30954374

[B15] Le Maitre C. L. Freemont A. J. Hoyland J. A. (2005). The role of interleukin-1 in the pathogenesis of human intervertebral disc degeneration. Arthritis Res. Ther. 7 (4), R732–R745. 10.1186/ar1732 15987475 PMC1175026

[B16] Le Maitre C. L. Pockert A. Buttle D. J. Freemont A. J. Hoyland J. A. (2007). Matrix synthesis and degradation in human intervertebral disc degeneration. Biochem. Soc. Trans. 35, 652–655. 10.1042/BST0350652 17635113

[B17] Leto Barone A. A. Khalifian S. Lee W. P. Brandacher G. (2013). Immunomodulatory effects of adipose-derived stem cells: Fact or fiction? Biomed. Res. Int. 2013, 1–8. 10.1155/2013/383685 PMC3782761 24106704

[B18] Liu W. Wang Y. Gong F. Rong Y. Luo Y. Tang P. (2019). Exosomes derived from bone mesenchymal stem cells repair traumatic spinal cord injury by suppressing the activation of A1 neurotoxic reactive astrocytes. J. Neurotrauma 36 (3), 469–484. 10.1089/neu.2018.5835 29848167

[B19] Loibl M. Wuertz-Kozak K. Vadalà G. Lang S. Fairbank J. Urban J. P. (2019). Controversies in regenerative medicine: Should intervertebral disc degeneration be treated with mesenchymal stem cells? JOR Spine 2 (1), e1043. 10.1002/jsp2.1043 31463457 PMC6711491

[B20] Madrigal M. Rao K. S. Riordan N. H. (2014). A review of therapeutic effects of mesenchymal stem cell secretions and induction of secretory modification by different culture methods. J. Transl. Med. 12, 260. 10.1186/s12967-014-0260-8 25304688 PMC4197270

[B21] Maffioli E. Nonnis S. Angioni R. Santagata F. Calì B. Zanotti L. (2017). Proteomic analysis of the secretome of human bone marrow-derived mesenchymal stem cells primed by pro-inflammatory cytokines. J. Proteomics 166, 115–126. 10.1016/j.jprot.2017.07.012 28739509

[B22] Mancuso P. Raman S. Glynn A. Barry F. Murphy J. M. (2019). Mesenchymal stem cell therapy for osteoarthritis: The critical role of the cell secretome. Front. Bioeng. Biotechnol. 7, 9. 10.3389/fbioe.2019.00009 30761298 PMC6361779

[B23] Matta A. Karim M. Z. Isenman D. E. Erwin W. M. (2017). Molecular therapy for degenerative disc disease: Clues from secretome analysis of the notochordal cell-rich nucleus pulposus. Sci. Rep. 7 (1), 45623. 10.1038/srep45623 28358123 PMC5372366

[B24] Mehrkens A. Matta A. Karim M. Z. Kim S. Fehlings M. G. Schaeren S. (2017). Notochordal cell-derived conditioned medium protects human nucleus pulposus cells from stress-induced apoptosis. Spine J. 17 (4), 579–588. 10.1016/j.spinee.2017.01.003 28089818

[B25] Parsons A. M. Ciombor D. M. Liu P. Y. Darling E. M. (2018). Regenerative potential and inflammation-induced secretion profile of human adipose-derived stromal vascular cells are influenced by donor variability and prior breast cancer diagnosis. Stem Cell Rev. Rep. 14 (4), 546–557. 10.1007/s12015-018-9813-1 29663271 PMC6014910

[B26] Petrenko Y. Syková E. Kubinová Š. (2017). The therapeutic potential of three-dimensional multipotent mesenchymal stromal cell spheroids. Stem Cell Res. Ther. 8 (1), 94. 10.1186/s13287-017-0558-6 28446248 PMC5406927

[B27] Risbud M. V. Shapiro I. M. (2014). Role of cytokines in intervertebral disc degeneration: Pain and disc content. Nat. Rev. Rheumatol. 10 (1), 44–56. 10.1038/nrrheum.2013.160 24166242 PMC4151534

[B28] Romaniyanto F. M. Prakoeswa C. R. S. Notobroto H. B. Tinduh D. Ausrin R. (2022). Hypoxia effects in intervertebral disc-derived stem cells and discus secretomes: An *in vitro* study. Stem Cells Cloning Adv. Appl. 15, 21–28. 10.2147/sccaa.S363951 PMC9153942 35655962

[B29] Russo F. De Salvatore S. Ambrosio L. Vadala G. Fontana L. Papalia R. (2021). Does workers' compensation status affect outcomes after lumbar spine surgery? A systematic review and meta-analysis. Int. J. Environ. Res. Public Health 18 (11), 6165. 10.3390/ijerph18116165 34200483 PMC8201180

[B30] Sakai D. Andersson G. B. (2015). Stem cell therapy for intervertebral disc regeneration: Obstacles and solutions. Nat. Rev. Rheumatol. 11 (4), 243–256. 10.1038/nrrheum.2015.13 25708497

[B31] Tilotta V. Vadalà G. Ambrosio L. Russo F. Cicione C. Di Giacomo G. (2021). Mesenchymal stem cell-derived exosomes: The new frontier for the treatment of intervertebral disc degeneration. Appl. Sci. 11 (23), 11222. 10.3390/app112311222

[B32] Vadalà G. Ambrosio L. Russo F. Papalia R. Denaro V. (2019). Interaction between mesenchymal stem cells and intervertebral disc microenvironment: From cell therapy to tissue engineering. Stem Cells Int. 2019, 1–15. 10.1155/2019/2376172 PMC7294366 32587618

[B33] Vadala G. Ambrosio L. Russo F. Papalia R. Denaro V. (2021). Stem cells and intervertebral disc regeneration overview-what they can and can't do. Int. J. Spine Surg. 15, 40–53. 10.14444/8054 34376495 PMC8092931

[B34] Vadalà G. Di Giacomo G. Ambrosio L. Cicione C. Tilotta V. Russo F. (2022). The effect of irisin on human nucleus pulposus cells: New insights into the biological crosstalk between the muscle and intervertebral disc. Spine (Phila Pa 1976) [Epub ahead of print]. 10.1097/brs.0000000000004488 36149858

[B35] Vadala G. Russo F. Ambrosio L. Di Martino A. Papalia R. Denaro V. (2015). Biotechnologies and biomaterials in spine surgery. J. Biol. Regul. Homeost. Agents 29, 137–147. 26652500

[B36] Vizoso F. Eiro N. Cid S. Schneider J. Perez-Fernandez R. (2017). Mesenchymal stem cell secretome: Toward cell-free therapeutic strategies in regenerative medicine. Int. J. Mol. Sci. 18 (9), 1852. 10.3390/ijms18091852 28841158 PMC5618501

[B37] Wang H. Tian Y. Wang J. Phillips K. L. E. Binch A. L. A. Dunn S. (2013). Inflammatory cytokines induce NOTCH signaling in nucleus pulposus cells. J. Biol. Chem. 288 (23), 16761–16774. 10.1074/jbc.M112.446633 23589286 PMC3675609

[B38] Williams R. J. Tryfonidou M. A. Snuggs J. W. Le Maitre C. L. (2021). Cell sources proposed for nucleus pulposus regeneration. JOR Spine 4 (4), e1175. 10.1002/jsp2.1175 35005441 PMC8717099

[B39] Wu A. March L. Zheng X. Huang J. Wang X. Zhao J. (2020). Global low back pain prevalence and years lived with disability from 1990 to 2017: Estimates from the global burden of disease study 2017. Ann. Transl. Med. 8 (6), 299. 10.21037/atm.2020.02.175 32355743 PMC7186678

[B40] Zeng X. Lin J. Wu H. Yu J. Tu M. Cheang L. H. (2020). Effect of conditioned medium from human umbilical cord-derived mesenchymal stromal cells on rejuvenation of nucleus pulposus derived stem/progenitor cells from degenerated intervertebral disc. Int. J. Stem Cells 13 (2), 257–267. 10.15283/ijsc20027 32587132 PMC7378895

